# HomeOSD: Appliance Operating-Status Detection Using mmWave Radar

**DOI:** 10.3390/s24092911

**Published:** 2024-05-02

**Authors:** Yinhe Sheng, Jiao Li, Yongyu Ma, Jin Zhang

**Affiliations:** Shenzhen Key Laboratory of Safety and Security for Next Generation of Industrial Internet, Research Institute of Trustworthy Autonomous Systems, Department of Computer Science and Engineering, Southern University of Science and Technology, Shenzhen 518055, China; 11930822@mail.sustech.edu.cn (Y.S.); lij@sustech.edu.cn (J.L.); 12232427@mail.sustech.edu.cn (Y.M.)

**Keywords:** Internet of Things, smart home, appliance operating-status detection, mmWave radar, vibration

## Abstract

Within the context of a smart home, detecting the operating status of appliances in the environment plays a pivotal role, estimating power consumption, issuing overuse reminders, and identifying faults. The traditional contact-based approaches require equipment updates such as incorporating smart sockets or high-precision electric meters. Non-constant approaches involve the use of technologies like laser and Ultra-Wideband (UWB) radar. The former can only monitor one appliance at a time, and the latter is unable to detect appliances with extremely tiny vibrations and tends to be susceptible to interference from human activities. To address these challenges, we introduce HomeOSD, an advanced appliance status-detection system that uses mmWave radar. This innovative solution simultaneously tracks multiple appliances without human activity interference by measuring their extremely tiny vibrations. To reduce interference from other moving objects, like people, we introduce a Vibration-Intensity Metric based on periodic signal characteristics. We present the Adaptive Weighted Minimum Distance Classifier (AWMDC) to counteract appliance vibration fluctuations. Finally, we develop a system using a common mmWave radar and carry out real-world experiments to evaluate HomeOSD’s performance. The detection accuracy is 95.58%, and the promising results demonstrate the feasibility and reliability of our proposed system.

## 1. Introduction

The rapid development of Internet of Things (IoT) technology [1,2,3] has led to increasing attention towards smart homes. One of the key functions of smart homes is appliance operating-status detection, which involves tasks such as measuring fan wind speeds or detecting the washing or drying of a washing machine. This capability offers numerous benefits, including estimating power consumption, providing alerts for improper usage, and identifying appliance anomalies. Several sensing technologies exist for detecting the operating status of appliances. A conventional approach involves using smart sockets to monitor and record the power consumption of each individual appliance to detect status [4,5,6]. However, this approach requires attaching a new sensor for each appliance, which can be intrusive, cumbersome, and impractical for larger homes with many appliances. Non-Intrusive Load Monitoring (NILM) is indeed a more efficient system for estimating the power consumption of individual devices [7,8,9,10]. It utilizes a single smart meter to measure the total power consumption of a household and then calculates the power consumption of each appliance. However, it can be challenging to detect simultaneous status changes in multiple appliances using only the total power consumption.

In addition to monitoring power consumption, researchers explore the use of vibration as an alternative method for detecting appliance operating status. For example, some studies employ laser Doppler vibrometers to capture the vibrations produced by appliances [11,12]. However, a limitation of this approach is its inability to simultaneously capture the vibrations of multiple appliances. Another approach integrating Ultra-Wideband (UWB) technology with visual sensing has been proposed [13]. However, it is limited by the wavelength of UWB signals, which renders it unable to detect the extremely tiny vibrations of appliances such as fridges. Furthermore, it fails to consider the potential impact of human activity on the detection accuracy.

Overall, research on detecting the operating status of electrical appliances can be categorized into two approaches: contact-based and non-contact-based. A contact-based method requires additional sensors to be integrated into the existing circuitry. Conversely, non-contact methods utilize sensors such as laser or UWB radar, which do not require any modification to the household wiring. However, non-contact approaches either lack the capability to simultaneously monitor multiple appliances or are vulnerable to interference from surrounding human activity. Therefore, our objective is to develop a non-contact system that is capable of simultaneously detecting the operating status of multiple electrical appliances without interference from human activity. We propose utilizing Millimeter Wave (mmWave) radar as a non-contact sensing technology. mmWave radar is known for its ability to detect multiple targets at different distances without physical contact. Previous research studies [14,15,16,17] demonstrate the capability of mmWave radar for detecting tiny vibrations.

However, there are several challenges that need to be addressed. Firstly, the vibration of appliances is tiny, making the mmWave signal changes caused by these vibrations easily distorted. This poses a challenge in accurately detecting and interpreting the subtle changes in the mmWave signal. Secondly, when detecting vibrating appliances, interference from other moving objects, such as people, can occur. This interference may affect the accuracy of the detection and introduce false readings or inconsistencies in the results. Overcoming this challenge requires developing robust algorithms and signal-processing techniques to distinguish between the vibrations of the appliances and other sources of movement. Thirdly, due to the instability of the electrical current and mechanical wear and tear, the frequency of appliance vibrations may undergo certain fluctuations, thus affecting the recognition results.

In this study, we propose HomeOSD, a system for detecting appliance operating status based on mmWave radar. To tackle the challenge of signal distortion, we propose a solution. In particular, we process the original mmWave signal and employ special periodic average calculations to remove various types of noise present in the signal. By applying these calculations, the signal quality can be enhanced and the system performance can be improved. To address the second challenge of interference from other moving objects, we propose a novel Vibration Intensity Metric, which is designed to estimate the intensity of vibration while disregarding the Doppler effect caused by other movements. By focusing specifically on the Vibration Intensity related to appliances, we aim to differentiate it from other sources of movement and minimize interference in the detection process. To tackle the third challenge, we propose an Adaptive Weighted Minimum Distance Classifier (AWMDC) that can mitigate the impact of fluctuations in appliance vibrations on classification results. By incorporating adaptive mechanism, the classifier can adapt to the fluctuations in appliance vibration frequencies.

We conduct comprehensive evaluations to assess the performance of HomeOSD in various scenarios. The results demonstrate an impressive average detection accuracy of 95.58% in real-world settings. This highlights HomeOSD’s ability to accurately detect the operating status of multiple appliances simultaneously, even when there is interference from human activities. Our contributions can be summarized as follows:

1. We propose a novel appliance operating-status detection system called HomeOSD, which utilizes mmWave radar technology. As far as we know, HomeOSD stands out as the first non-contact system capable of simultaneously monitoring the operating status of multiple appliances without interference from human activity. This advancement significantly enhances smart sensing capabilities for home environments;
2. We propose a novel metric called Vibration Intensity Metric for detecting vibrating objects and design an AWMDC classifier. The Vibration Intensity Metric effectively mitigates interference from human activities, while AWMDC can adapt to the fluctuations in appliance vibration frequencies, enhancing the precision of appliance operating-status detection;
3. We implement our system using a commercial mmWave radar and conduct a thorough evaluation of its performance in various environments. The experimental results demonstrate the impressive accuracy of 95.58% achieved by HomeOSD for operating-status detection in real-world scenarios.

The remainder of this paper is as follows. In Section 2, a review of related works is provided. Section 3.1 presents an overview of the proposed system. The detailed design of the system, including mmWave Signal Pre-Processing, Vibrating Object Detection, and Operating-Status Identification, is presented in Section 3.2 through Section 3.4. Section 4 describes the implementation of the system and presents the results of the system evaluation. In Section 5, the findings and limitations of the study are discussed, while Section 6 provides a conclusion to the paper.

## 2. Related Works

This section reviews existing works on appliance operating-status detection and vibration measurement.

### 2.1. Appliance Operating-Status Detection

According to different sensing methods, existing home-appliance operating-status detection systems can be divided into two main categories: contact sensing-based and contactless sensing-based.

**Contact Sensing-Based:** The smart socket is a commonly used device that can record the power consumption of an appliance and transmit it to the smart-home network for analysis of the appliance’s status [4,5,6]. However, installing a smart socket for each appliance can be intrusive and expensive. In addition to the smart socket, NILM is a system to detect the appliance operating status by leveraging a single power meter to record the household’s consumption and extract the status change of each electrical appliance [7,8,9,10]. However, NILM can only capture the total consumption, making it difficult to detect the status of multiple appliances changing simultaneously.

**Contactless Sensing Based:** Laser vibrometry is a non-contact sensing technology, used to measure tiny displacements. By measuring the tiny vibrations of an appliance, it can detect its operating status [11,12]. Vibrosight [11] uses a laser vibrometer to measure the vibration of a tag attached to an appliance and identify its operating status. However, since a laser vibrometer can only measure the vibration of a single point, it requires scanning an entire room to detect all appliances. VibroSense [12] uses a laser vibrometer to measure the vibration of the wall instead of directly measuring the vibration of appliances to detect their status. However, when many appliances are running simultaneously, it can be difficult to extract the vibrations of all electrical appliances through the vibration of a single point on the wall. Additionally, laser vibrometers cannot be used for long periods, as they are harmful to human eyes, making continuous monitoring challenging. Capricorn incorporates UWB technology combined with visual sensing to monitor the status of electrical appliances [13]. However, UWB’s operation is constrained by its inherent wavelength limitations, ranging from 28.3 mm to 96.8 mm, which preclude detection of minuscule vibrations such as those generated by appliances like refrigerators. In addition, Capricorn neglects to consider the influence of human activities on appliance operating-status detection.

To the best of our knowledge, in scenarios involving human activity interference, there is currently no contactless system that can simultaneously detect the operating status of multiple appliances based on tiny vibrations. Therefore, we aim to go a step further and extend vibration detection of a single object to vibration detection of multiple objects simultaneously.

### 2.2. Vibration Measurement

Current vibration measurement technology can be divided into three main categories: contact sensor-based, optical-based, and Ratio Frequency (RF)-based.

**Contact Sensor-Based:** Vibration can be detected by attaching contact sensors to the target surface [18,19,20]. For example, piezoelectric sensors are a type of contact sensor that measure vibration by sensing the force changes on the sensors caused by vibration. The principle of vibration detection based on piezoelectric sensors is to mount the sensor on a target surface. When the surface vibrates, the piezoelectric sensor senses the force change caused by the vibration and converts it into an electrical signal output. This signal’s amplitude and frequency can be used to analyze the target surface’s vibration state. However, the use of contact sensors may increase the cost of deployment and maintenance.

**Optical-Based:** Laser vibrometers use the Doppler effect to detect tiny vibrations [21,22,23,24]. They have high measurement accuracy due to the short wavelength of the laser. However, high-precision laser vibrometers are expensive and can only measure the vibration of one point at a time. On the other hand, high-speed cameras are widely used for vibration monitoring [25,26,27,28]. However, cameras are vulnerable to ambient light and cause privacy issues, making them unsuitable for detecting appliance operating status.

**RF-Based:** In recent years, several solutions for detecting vibrations based on RFID have been proposed [29,30,31,32,33,34]. By attaching a tag, the vibration of the target surface can be detected. RFID has the advantage of being low-cost and not depending on lighting conditions. However, due to the large wavelength of RFID, its accuracy in measuring tiny vibrations is limited. Alternatively, the Impulse Radio Ultra-Wideband (IR-UWB) can be used to detect vibrations. The frequency of IR-UWB ranges from 3.1 to 10.6 GHz [35], which allows it to penetrate walls and measure vital signs [36,37,38,39] and the vibration of speakers [40]. However, its ability to detect tiny vibrating objects such as a running fridge is limited [13], similar to RFID. In comparison, mmWave radar has a shorter wavelength and can detect tiny displacements. It can capture fine-grained vital signs [41,42]. Since sound is usually produced by tiny vibrations, many works utilize mmWave radar to detect the vibration caused by sound [15,43,44,45,46]. Meanwhile, utilizing the penetrative capability of mmWave radar, many works use it to detect minor vibrations for eavesdropping [45,47,48,49,50,51]. In addition, mmWave radar is employed in detecting minute vibrations for mechanical fault detection [16,17,52], emerging communications [14], and material identification [53]. Furthermore, common mmWave radar, such as Frequency-Modulated Continuous-Wave (FMCW) radar, can effortlessly extract the minute displacements of multiple targets simultaneously [54,55,56].

Given the advantages of mmWave radar, such as high precision, contactless sensing, multi-target detection, and the characteristic that it is unaffected by ambient light, mmWave radar is more suitable for detecting vibrations of electrical appliances. Therefore, we utilize the mmWave radar to implement our design.

## 3. HomeOSD System Design

### 3.1. System Overview

**Figure 1:**
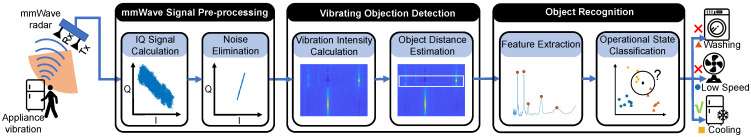
System overview of HomeOSD.

Our system, HomeOSD, consists of three primary modules: mmWave Signal Pre-Processing, Vibrating-Object Detection, and Operating-Status Identification. As illustrated in Figure 1, the beat frequency signal from the mmWave radar is input to the mmWave Signal Pre-Processing module to calculate the IQ components and eliminate the noise. Then, the high-quality signal is fed into the Vibrating-Object Detection module to conduct the Vibration Intensity calculation and object distance estimation. After the object distance estimation, the Operating-Status Identification module extracts features from the Vibration Intensity spectrum and identifies the operating status of the detected objects.

**mmWave Signal Pre-Processing:** This module is designed to eliminate the noise in the original signal. Since the vibration of electrical appliances is weak, noise in the signal can significantly impact the accuracy of detection. To address this issue, we utilize several Signal Pre-Processing techniques to eliminate noise and produce a high-quality signal.

**Vibrating-Object Detection:** After pre-processing, the signal is input to this module to detect the vibrating objects. The challenge lies in that the Doppler effect due to moving objects can obscure the Doppler effect of appliance vibration, making it easy to misinterpret vibration detection. To address this issue, we propose a novel Vibration Intensity Metric based on periodicity. By calculating the Vibration Intensity of objects at various distances, we can detect the distance of each vibrating object from the radar without interference from other moving objects.

**Operating-Status Identification:** When the vibrating objects are detected, this module extracts features from the Vibration Intensity spectrum. Due to the fluctuating frequency of appliance vibrations resulting from electrical current instability and mechanical wear, we develop an AWMDC. This classifier can dynamically adapt to these vibration frequencies fluctuating, ensuring accurate classification of appliance operating status.

### 3.2. Signal Pre-Processing

This section describes the mmWave Signal Pre-Processing module in our system, which aims to improve signal quality by removing noise from the raw mmWave radar signal.

#### 3.2.1. IQ Signal Calculation

We use a Frequency-Modulated Continuous-Wave (FMCW) mmWave radar to detect the slight vibrations of objects. The radar emits a continuous chirp signal that is linearly modulated in frequency:

(1) $$s_{\mathrm{Tx}}\left(t\right)=\exp\left[j\left(2\pi f_{c}t+\pi Kt^{2}\right)\right],t\in \left[0,T\right],$$

where $s_{\mathrm{Tx}}$ is the radar’s Transmitted Signal (Tx), $f_{c}$ is the starting frequency, *K* is the slope of the chirp signal, *T* is the period of the chirp, and time *t* ranges from 0 to *T*. Objects in the vicinity reflect the transmitted signals, and the radar receives the sum of these reflected signals. The Received Signal (Rx) can be expressed as follows:

(2) $$s_{\mathrm{Rx}}\left(t\right)=\sum_{i}\alpha_{i}s_{\mathrm{Tx}}\left(t-\frac{2d_{i}}{c}\right),$$

where $\alpha_{i}$ represents the path loss of the signal reflected by the *i*-th object, $d_{i}$ is the distance of the *i*-th object, and *c* is the speed of the signal. The Rx signal is mixed with the Tx signal and passed through a low-pass filter, and the output is the beat frequency signal s(t):

(3) $$\begin{array}{cc} s(t) & =s_{\mathrm{Tx}}\left(t\right)s_{\mathrm{Rx}}^{*}\left(t\right)\\ \; & \approx \sum_{i}\alpha_{i}\exp\left[j\cdot 2\pi \left(K\frac{2d_{i}}{c}t+\frac{2d_{i}}{\lambda }\right)\right], \end{array}$$

where λ is the wavelength of the signal. The frequency response of the time-domain signal can be calculated using the Fourier Transform:

(4) $$\begin{array}{cc} S(f) & =\int_{0}^{T}s\left(t\right)\cdot \left(-j\cdot 2\pi ft\right)dt\\ \; & =\sum_{i}A_{i}\exp\left(j\cdot 2\pi \frac{2d_{i}}{\lambda }\right), \end{array}$$

where *f* is the subcarrier of the frequency response, $A_{i}$ is the amplitude of the component of the *i*-th object, and $A_{i}=\alpha_{i}T\cdot sinc\left[\left(\frac{2d_{i}}{c}K-f\right)T\right]\cdot \exp\left(j\cdot \pi \left(\frac{2d_{i}}{c}K-f\right)T\right)$. When $d_{i}$ is close to $\frac{cf}{2K}$, the $A_{i}$ is close to 1; otherwise, it is close to 0. In practical scenarios, the surrounding reflectors include both moving and stationary objects. The radar continuously transmits sweeps of the chirp, and the distance $d_{i}$ of the moving object is different in each sweep. Therefore, we can rewrite Equation (4) as follows:

(5) $$\begin{array}{cc} S(k,f) & =\sum_{p}A_{p}\exp\left(j\cdot 2\pi \frac{2d_{p}\left(k\right)}{\lambda }\right)+\sum_{q}A_{q}\exp\left(j\cdot 2\pi \frac{2d_{q}\left(k\right)}{\lambda }\right)\\ \; & =\sum_{p}A_{p}\exp\left(j\cdot 2\pi \frac{2d_{p}\left(k\right)}{\lambda }\right)+A_{0}\exp\left(j\cdot 2\pi \frac{2d_{0}}{\lambda }\right), \end{array}$$

where *k* corresponds to the *k*-th sweep of the chirp, *p* corresponds to the *p*-th moving object, and *q* corresponds to the *q*-th stationary object. $A_{0}$ and $d_{0}$ represent the virtual amplitude and distance, respectively, of all stationary objects. By using Euler’s formula, we can obtain the signal in the IQ domain:

(6) $$\begin{array}{cc} I(k,f) & =real\left(S(k,f)\right)\\ \; & =\sum_{p}A_{p}\cos\left(2\pi \frac{2d_{p}\left(k\right)}{\lambda }\right)+A_{0}\cos\left(2\pi \frac{2d_{0}}{\lambda }\right), \end{array}$$

(7) $$\begin{array}{cc} Q(k,f) & =imag(S(k,f))\\ \; & =\sum_{p}A_{p}\sin\left(2\pi \frac{2d_{p}\left(k\right)}{\lambda }\right)+A_{0}\sin\left(2\pi \frac{2d_{0}}{\lambda }\right). \end{array}$$

Based on Equations (5)–(7), we define the stationary component of the vibrating object, the moving component of the vibrating object, and the total signal s(f,k) in the IQ domain as the static vector $\vec{S_{S}}$, the dynamic vector $\vec{S_{D}}$, and combined vector $\vec{S_{C}}$, respectively.

**Figure 2:**
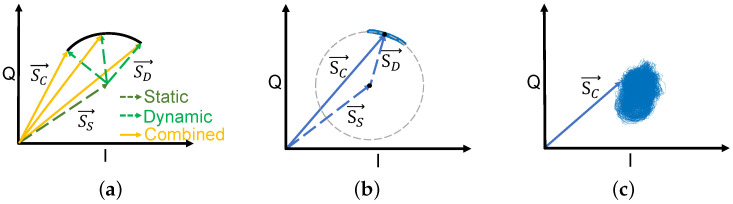
Illustration of different vibrating objects in the IQ domain. (**a**) Ideal points and (**b**) vibration generator show arc-shaped trajectories, while (**c**) the trajectory of the air conditioner is highly chaotic.

Ideally, when there is only one vibrating object in the vicinity, the length of the dynamic vector $\vec{S_{D}}$ remains constant, and the trajectory of $\vec{S_{C}}$ in the IQ domain takes the shape of an arc, as depicted in Figure 2a. To validate the model, we employ a vibration generator to simulate the vibration and detect it in an environment without human activity. As shown in Figure 2b, the signal in the IQ domain conforms to the model. However, in real scenarios where appliance operating status detection is required, there may exist various vibrating objects and people walking around. As a result, the length of the dynamic vector $\vec{S_{D}}$ may not be constant, and the trajectory of $\vec{S_{C}}$ in the IQ domain may not be an arc. As shown in Figure 2c, the signal $\vec{S_{C}}$ from a real-world scenario where an air conditioner is running is no longer an arc, but a more complex shape.

Above observation indicates that the effects of surrounding vibrating objects and human activities cannot be ignored. However, previous works on vibration detection based on mmWave radar have only considered the situation shown in Figure 2a. Therefore, we aim to propose a system that can detect multiple vibrating objects in more complicated real-world scenarios.

#### 3.2.2. Noise Elimination

During the process of detecting the operating status of appliances, signal noise primarily arises from random noise and baseline drift. Random noise refers to a signal that contains random fluctuations and does not exhibit any discernible pattern or structure [57,58]. The presence of random noise in many applications is often undesirable, as it can interfere with the clarity and accuracy of desired signals or data. In radar systems used for detecting vibrations, random noise can cause significant interference to the system, particularly as the amplitude of the vibration of the object being measured is typically relatively small [16,17]. In addition, baseline drift can be caused by the radar itself or by the surroundings [59]. Human activities also produce periodic movements. Most of these are large in amplitude, such as walking and hand-waving. In addition, there are some small amplitude movements such as the chest movements caused by the heartbeat. But these small-amplitude periodic motions are also much lower in frequency than the vibrations of electrical appliances, so any signal changes caused by human activity are equivalent to drift noise. Therefore, our noise elimination design comprises two parts: random-noise elimination and baseline-drift elimination.

**Random-noise elimination:** Random noise is an inherent part of any system and can be effectively eliminated through applying moving average filter to the signal in time domain. Specifically, the moving average process replaces the value of each point with the average value of its neighboring points:

(8) $$S_{1}\left(k,f\right)=\frac{1}{M_{1}}\sum_{i=-\frac{M_{1}}{2}}^{\frac{M_{1}}{2}}S\left(k+i,f\right),$$

where $S_{1}\left(k,f\right)$ is the signal after the moving average process and $M_{1}$ is the number of points in the moving window. If the moving window is too small, its ability to reduce random noise through moving averaging will be insufficient. Conversely, when the moving window approaches or exceeds the period of a periodic signal, significant components of that signal will be attenuated. Therefore, assuming our target vibration period to be $T_{a}$, we use a moving window length equal to half the length of period $T_{a}$. The value assigned to $M_{1}$ corresponds to the number of sampling points associated with $T_{a}/2$.

**Figure 3:**
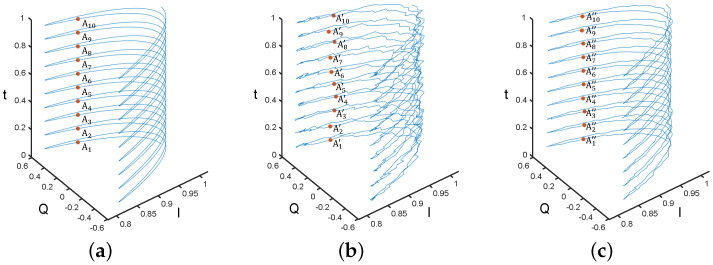
(**a**) Ideal periodic signal in the IQ domain; (**b**) Periodic signal with random noise in the IQ domain; (**c**) Signal obtained by specific averaging of the signal in (**b**). From point $A_{1}$ to point $A_{10}$, from point $A_{1}^{\prime }$ to point $A_{10}^{\prime }$, and from point $A_{1}^{\prime \prime }$ to point $A_{10}^{\prime \prime }$, the time interval is a period.

Compared to other signals, periodic signals have a distinct characteristic: the points on the ideal periodic signal return to the same value after an integer multiple of periods, as exemplified by points $A_{1}$ to $A_{10}$ in Figure 3a. Therefore, random noise can be further eliminated by averaging points whose time interval is the period, as shown in Figure 3b,c. However, it is necessary to first assume various vibration periods and calculate the noise-eliminated signal under each of these assumed periods. The process is expressed by the following equation:

(9) $$S_{2}\left(k,f\right)=\frac{1}{M_{2}}\sum_{i=-\frac{M_{2}}{2}}^{\frac{M_{2}}{2}}S_{1}\left(k+i\cdot T_{a},f\right),$$

where $S_{2}\left(k,f\right)$ represents the signal obtained by averaging $M_{2}$ points with the assumed vibration period $T_{a}$. Similar to $M_{1}$ in Equation (8), an excessively small $M_{2}$ can also hinder the ability to suppress random noise effectively. Conversely, when the length of signal $S_{2}$ is fixed, an overly large $M_{2}$ would result in an output $M_{2}$ of insufficient length following Equation (9). Taking into account the sampling rate, length, and range of $T_{a}$ for our signals, we have chosen $M_{2}$ to be 5 within our system.

**Figure 4:**
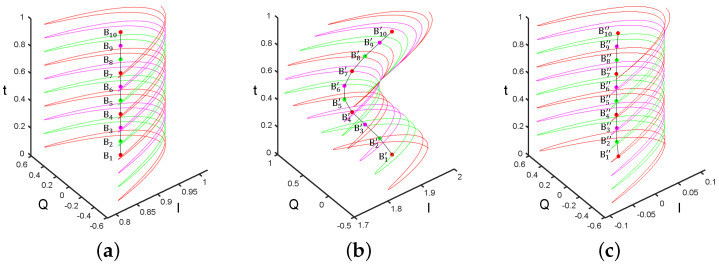
(**a**) Ideal periodic signal and its mean value for each period in the IQ domain; (**b**) Periodic signal with baseline drift and its mean value for each period in the IQ domain; (**c**) Signal obtained after baseline-drift removal from the signal in (**b**).

**Baseline-drift elimination:** In a noise-free periodic signal, the average value of each period of a periodic signal remains constant, as shown from points $B_{1}$ to $B_{10}$ in Figure 4a. However, when the periodic signal contains drift, the average value of each period can reflect its component of drift, as shown by points $B_{1}^{\prime }$ to $B_{10}^{\prime }$ in Figure 4b. Therefore, an approximate drift component can be obtained by using the moving average of a window with the length of the period. Finally, we subtract this component from the original signal to eliminate the drift, as shown in Figure 4c. The equation for this process is written as

(10) $$S_{3}\left(k,f\right)=S_{2}\left(k,f\right)-\frac{1}{T_{a}}\sum_{i=-\frac{T_{a}}{2}}^{\frac{T_{a}}{2}}S_{2}\left(k+i,f\right),$$

where $S_{3}\left(k,f\right)$ is the signal after baseline-drift elimination.

To process the signal of an electrical appliance, it is necessary to assume all possible frequencies, as the frequency of vibration is unknown beforehand.

**Figure 5:**
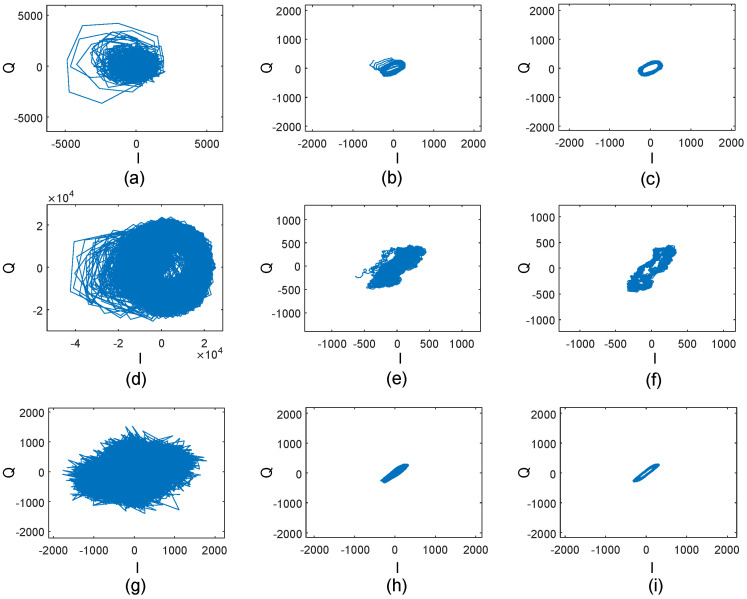
(**a**–**c**), (**d**–**f**), and (**g**–**i**) show the IQ domain signals of the air conditioner, fan, and refrigerator, respectively. (**a**,**d**,**g**) represent the original signals. (**b**,**e**,**h**) represent the signals after the elimination of random noise. Finally, (**c**,**f**,**i**) represent the signals after the elimination of both random noise and baseline drift.

Figure 5 illustrates the raw signals, signal after random noise elimination, signal after random-noise elimination, and baseline-drift elimination for three appliances. We can observe that the signals after the random-noise removal exhibit significantly improved signal-to-noise ratios compared to the raw signals, and the signals after both random-noise removal and baseline-drift removal are much clearer. The results demonstrate the effectiveness of our proposed approach.

### 3.3. Vibrating-Object Detection

#### 3.3.1. Vibration Intensity Calculation

Previous studies [16,17] have typically utilized the Doppler effect to detect vibrating objects. However, in indoor environments, the Doppler effect from human motion is often much larger than that from appliance vibration. Therefore, in real-world environments, the Doppler effect from appliance vibration may be masked.

**Figure 6:**
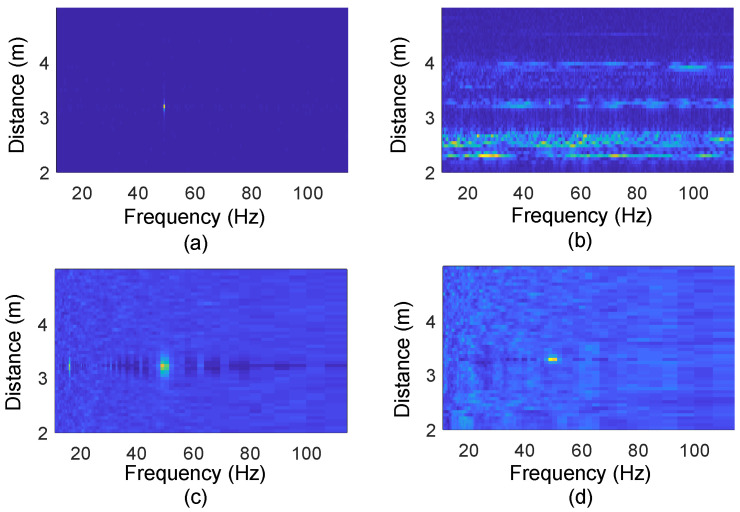
Range-Doppler spectrum under scenarios where there is (**a**) no interference and (**b**) one person walking around; Vibration Intensity spectrum under scenarios where there is (**c**) no interference and (**d**) one person walking around.

Figure 6a,b demonstrates the Doppler spectrum obtained from vibration detection of a refrigerator with radar in the absence of any interference and in a scenario where someone is walking around, respectively. It is obvious that the energy caused by the walking person masks and drowns out the energy corresponding to the vibration of the refrigerator. Consequently, a new metric that allows robust vibration detection against interference needs to be proposed.

**Figure 7:**
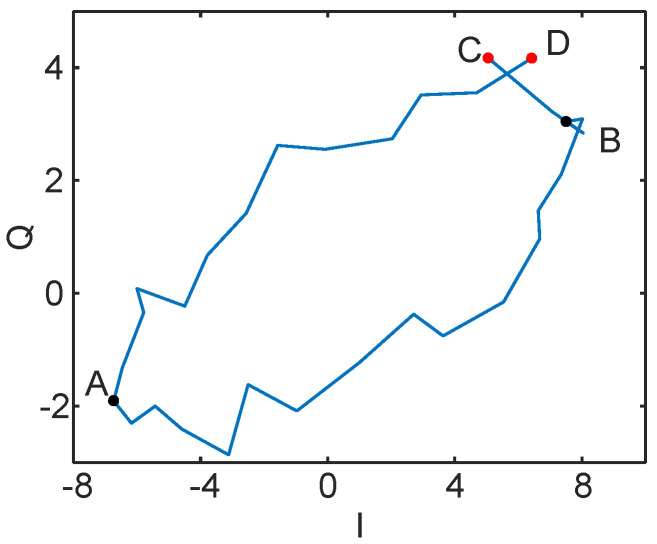
One period of a vibration signal in the IQ domain. *A* and *B* are two points with a time interval of half a period and *C* and *D* are two points with a time interval of one period.

For the periodic signal, we observe that the distance between two points in the IQ domain is related to the signal’s period. Specifically, the time interval between the farthest points is half a period, and the time interval between the closest points is one period. We also verify this characteristic of the periodic signal in practice, as shown in Figure 7. The time interval between *A* and *B* is half a period, and the time interval between *C* and *D* is one period. For points within a period, we can express the farthest distance $D_{f}$ and the average nearest distance $D_{n}$ using the following equations:

(11) $$D_{f}\left(k_{0},T_{a}\right)=\max_{k_{0}<k<T_{a}+k_{0}}\left\{\left|\left(S_{3}\left(k+\frac{T_{a}}{2},f\right)-S_{3}\left(k,f\right)\right)\right|\right\},$$

(12) $$D_{n}\left(k_{0},T_{a}\right)=\underset{k_{0}<k<T_{a}+k_{0}}{avg}\left\{\left|\left(S_{3}\left(k+T_{a},f\right)-S_{3}\left(k,f\right)\right)\right|\right\},$$

where $T_{a}$ is the assumed period of the vibration and $k_{0}$ is the first point in current period that is computed.

By substituting the assumed vibration frequency $f_{a}$ for $T_{a}$ in Equations (11) and (12), we can obtain $D_{f}\left(k_{0},f_{a}\right)$ and $D_{n}\left(k_{0},f_{a}\right)$ as

(13) $$D_{f}\left(k_{0},f_{a}\right)=\max_{k_{0}<k<\frac{1}{f_{a}}+k_{0}}\left\{\left|\left(S_{3}\left(k+\frac{1}{2f_{a}},f\right)-S_{3}\left(k,f\right)\right)\right|\right\},$$

(14) $$D_{n}\left(k_{0},f_{a}\right)=\underset{k_{0}<k<\frac{1}{f_{a}}+k_{0}}{avg}\left\{\left|\left(S_{3}\left(k+\frac{1}{f_{a}},f\right)-S_{3}\left(k,f\right)\right)\right|\right\}.$$

Since each $S_{3}$ signal usually contains multiple vibration periods, to fully utilize the information of the entire signal, we can calculate the average values of $D_{f}\left(f_{a}\right)$ and $D_{n}\left(f_{a}\right)$ over the entire signal:

(15) $${\bar{D}}_{f}\left(f_{a}\right)=\underset{0<k_{0}<k_{n}-1/f_{a}}{avg}\left\{D_{f}\left(k_{0},f_{a}\right)\right\},$$

(16) $${\bar{D}}_{n}\left(f_{a}\right)=\underset{0<k_{0}<k_{n}-1/f_{a}}{avg}\left\{D_{n}\left(k_{0},f_{a}\right)\right\},$$

where $k_{n}$ is the chirp number of the entire signal. We observe that, when $1/f_{a}$ is closer to the real vibration period, the value of ${\bar{D}}_{f}\left(f_{a}\right)$ increases while the value of ${\bar{D}}_{n}\left(f_{a}\right)$ decreases. Therefore, we can leverage the ratio of their values to reflect the intensity of vibration at different frequencies:

(17) $$VI\left(f_{a}\right)=\frac{{\bar{D}}_{f}\left(f_{a}\right)}{{\bar{D}}_{n}\left(f_{a}\right)},$$

where we define $VI(f_{a})$ as the Vibration Intensity Metric.

**Figure 8:**
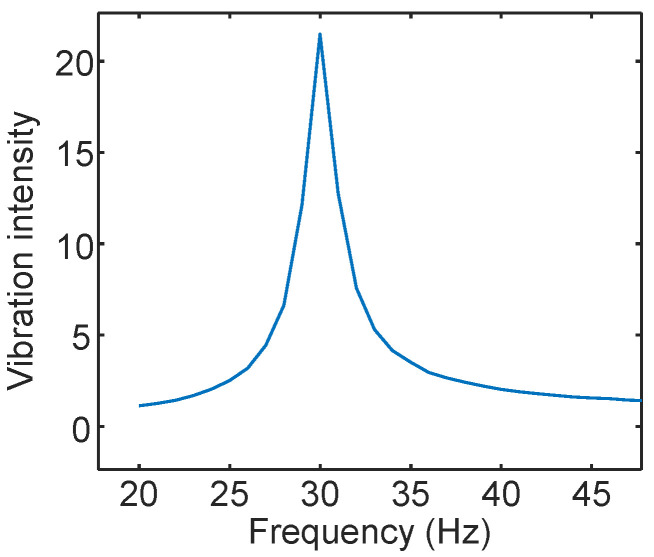
Vibration Intensity of a 30 Hz vibration object at different frequencies.

Figure 8 shows the Vibration Intensity of a 30 Hz vibrating object, as calculated from the reflected radar signal. It exhibits a clear peak at 30 Hz, indicating the effectiveness of the proposed metric in evaluating the IQ signal’s intensity at various vibration frequencies.

We compare the Range–Doppler spectrum and Vibration Intensity spectrum of a fridge in two different scenarios when nobody is around and when someone is walking around, as shown in Figure 6. It can be observed that only the Vibration Intensity remains consistent in both cases (Figure 6c,d), while the Doppler effect experiences significant changes due to the influence of the person walking (Figure 6a,b).

#### 3.3.2. Object-Distance Estimation

**Figure 9:**
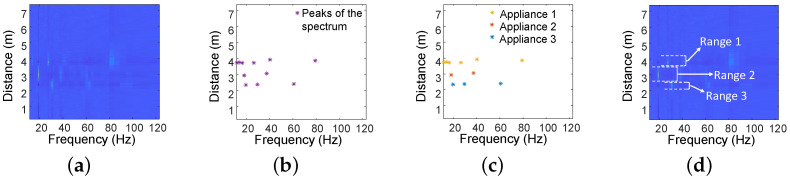
To estimate the range of appliances from (**a**) the Vibration Intensity spectrum, the process involves three steps: (**b**) first, extract peaks from the spectrum; (**c**) then, cluster to identify different appliances; and (**d**) finally, extract the range for each appliance.

In this section, we utilize the Vibration Intensity Metric to estimate the distances of all vibrating objects. Figure 9a illustrates the Vibration Intensity spectrum in distance and frequency for three electrical appliances. Notably, each appliance exhibits distinctive peaks at corresponding distances, which we can leverage to estimate the distances of all appliances. The overall algorithm is depicted in Figure 9 and Algorithm 1.
**Algorithm 1:** Object-distance estimation algorithm
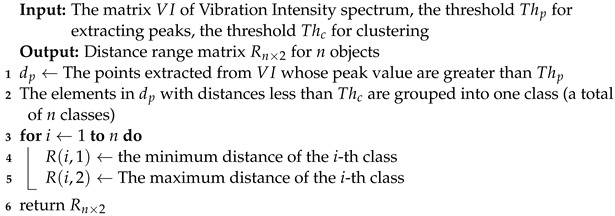


### 3.4. Operating-Status Identification

In particular, we firstly identify peaks across the entire Vibration Intensity spectrum, retaining only those that exceed a pre-set threshold (Algorithm 1 line 1). Then, we group the peaks within a specific distance range into the same appliance cluster (Algorithm 1 line 2). Finally, we consider the minimum and maximum distances of the peak points in each cluster as the distance range for that appliance (Algorithm 1 line 3 to 5). The distance range of each cluster will be input to the next module.

As shown in Figure 6, the Vibration Intensity spectrum is significantly more stable than the Doppler spectrum in different environments. Therefore, we extract features that are related to the Vibration Intensity. Then, we use these Vibration Intensity features to classify the operating status of the appliance.

#### 3.4.1. Feature Extraction

**Figure 10:**
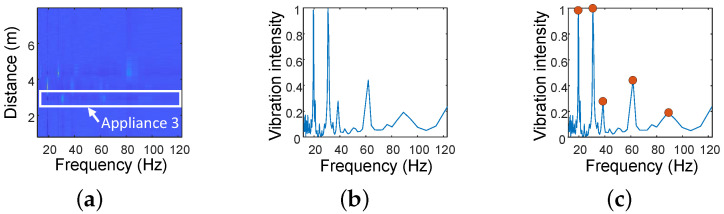
The process of feature extraction: (**a**) extract the Vibration Intensity spectrum within the range of each appliance; (**b**) extract the strongest Vibration Intensity at each frequency; (**c**) select the top-*k* optimal vibration frequencies; and, finally, take the optimal top-*k* frequencies along with their corresponding vibration intensities as the features for classification.

In this section, we describe the process of feature extraction for appliance operating-status classification. As shown in Figure 6d, even in the presence of human-activity interference, the frequencies detected using the Vibration Intensity Metric remain stable. Therefore, we extract these frequencies and their Vibration Intensities as features for classification. The feature extraction process consists of three steps, as illustrated in Figure 10. The detailed process is also shown in Algorithm 2. Firstly, we extract the Vibration Intensity spectrum of the target appliance within its distance range (Algorithm 2, line 1). Then, we extract the maximum Vibration Intensity for each frequency within the distance range of the respective appliance (Algorithm 2, line 2). Finally, we select the top *k* frequencies with the maximum normalized Vibration Intensity (Algorithm 2, lines 3 to 8). These identified top-*k* frequencies, along with their corresponding Vibration Intensities, are regarded as the final extracted features for classification.
**Algorithm 2:** Feature-extraction algorithm
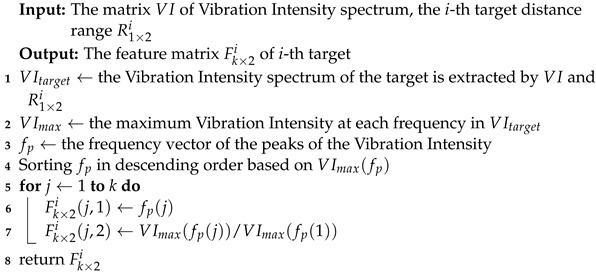


#### 3.4.2. Operating-Status Classification

As the features extracted based on Vibration Intensity are stable, there is no need to employ complex machine learning methods for classification. After comparing different approaches, we decided to use a Weighted Minimum Distance Classifier (WMDC) [60] to identify the status of appliances. The fundamental concept of WMDC involves comparing the distances between test samples and training samples of different types and, subsequently, assigning the type of training sample with the smallest distance as the classification for the test sample. Due to fluctuations caused by current variations or mechanical wear and tear, the vibration frequency may change slightly, even within the same appliance’s operating status, which can cause misjudgment. To tackle this, we devise an Adaptive Weighted Minimum Distance Classifier (AWMDC) that can reduce the impact of vibration frequency fluctuations.

Like traditional WMDC classifiers, our AWMDC method determines the class of a test sample by calculating its proximity to the training sample:

(18) $$\hat{i}=\min_{1\leq i\leq n}d_{i}\left(\hat{j},\hat{\mu }\right),$$

where $\hat{i}$ represents the determined appliance operating status, $d_{i}$ signifies the distance between the training sample of the *i*-th class and the test sample, and *n* corresponds to the total number of appliance operating statuses. In this section, we present two adaptive strategies aimed at enhancing the classifier’s robustness:

- **Adaptive Feature Selection:** We dynamically choose a frequency combination to ensure that the selected feature set effectively represents the distribution of the current category for subsequent classification. Here, $\hat{j}$ represents the chosen *j*-th frequency combination;
- **Relaxation Coefficient:** We introduce a relaxation coefficient μ to accommodate variations in vibration frequencies. $\hat{\mu }$ denotes the selected relaxation coefficient, adapting to the specific conditions.

The vibration of different surfaces of the same appliance is different. Therefore, it is necessary to dynamically select representative frequency features for distance calculation. Out of the total number of extracted features containing *k* frequencies, we choose to retain only *m* frequencies. This yields potential combinations that total $C_{k}^{m}$. The criterion for selecting *j* is to minimize the value of $d_{i}\left(j,\hat{\mu }\right)$ to ensure that the set of selected features, i.e., the frequency combinations, are highly representative:

(19) $$\hat{j}=\min_{1\leq j\leq C_{k}^{m}}d_{i}\left(j,\hat{\mu }\right).$$

The specific expression for $d_{i}\left(j,\hat{\mu }\right)$ is

(20) $$d_{i}\left(j,\hat{\mu }\right)=\left\{\sum_{p=1}^{m}[{\left(f_{j}^{\prime }\left(p\right)-\hat{\mu }f_{j}\left(i,p\right)\right)}^{2}+\omega_{0}{\left(VI_{j}^{\prime }\left(p\right)-VI_{j}\left(i,p\right)\right)}^{2}\right]{\}}^{\frac{1}{2}},$$

where $f_{j}^{\prime }\left(p\right)$ and $VI_{j}^{\prime }\left(p\right)$ correspond to the *p*-th frequency and Vibration Intensity, respectively, within the *j*-th frequency combination among the features of the test sample. Likewise, $f_{j}\left(i,p\right)$ and $VI_{j}\left(i,p\right)$ denote the *p*-th frequency and Vibration Intensity, respectively, within the *j*-th frequency combination among the average features of the training sample of the *i*-th class. Additionally, $\omega_{0}$ represents a constant weighting coefficient.

The introduction of the relaxation coefficient μ aims to mitigate the impact of vibration frequency fluctuations. Similar to the criterion for selecting *j*, we calculate μ by minimizing $d_{i}\left(j,\mu \right)$ for the *j*-th frequency combination:

(21) $$\hat{\mu }=\min_{\mu_{1}\leq \mu \leq \mu_{2}}\left\{\sum_{p=1}^{m}[{\left(f_{j}^{\prime }\left(p\right)-\mu f_{j}\left(i,p\right)\right)}^{2}+\omega_{0}{\left(VI_{j}^{\prime }\left(p\right)-VI_{j}\left(i,p\right)\right)}^{2}\right]{\}}^{\frac{1}{2}},$$

where $\mu_{1}$ and $\mu_{2}$ represent the lower and upper bounds of μ, respectively.

While the aforementioned design enhances classifier robustness, our system encounters a challenge when simultaneously testing multiple appliances: those with significant vibration amplitudes may overshadow those with smaller amplitudes. Moreover, real-world scenarios can involve human movements that obstruct the radar’s propagation path to appliances. To tackle these issues, we employ a strategy where we aggregate results from *N* data segments, each of length *l* seconds, and the sliding window stride for each segment is *s* seconds (with *l* set to 2.4 s, *s* set to 1 s, and *N* fixed at 10 s). Therefore, each segment evaluation requires 11.4 s of data, with a 1 s interval between each identification. This approach effectively compensates for undetected appliances and their corresponding operating statuses.

## 4. Evaluation

### 4.1. Experimental Setup

**Figure 11:**
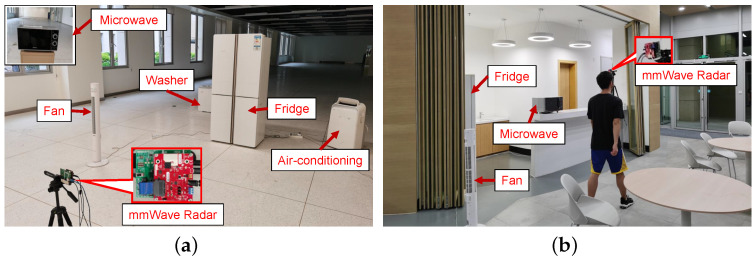
Hardware and experimental scenarios. (**a**) Office room; (**b**) Real-world scenario.

**Hardware and Software:** As shown in Figure 11a, our testbed is a commercial mmWave radar, TI IWR1443 BoosterPack, with a bandwidth of 4 GHz (77∼81 GHz) and a total of seven antennas (three Tx antennas and four Rx antennas). We use one Tx antenna to send FMCW signals with a bandwidth of 2.5 GHz and all Rx antennas to receive reflected signals. The raw signals are sampled at 35 MHz and the chirp signals are sampled at 8 kHz. The raw signals of the radar are acquired by a TI DCA1000EVM data acquisition card. All algorithms in our system are implemented in Matlab on a computer equipped with an AMD Ryzen 7 3700X processor and 16 GB of RAM.

**Figure 12:**
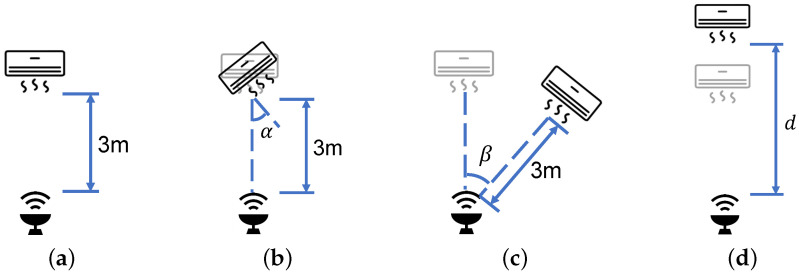
Position relationship between radar and electrical appliance. (**a**) Facing each other; (**b**) Different orientations; (**c**) Different AoAs; (**d**) Different distances.

**Data Collection:** We test 5 appliances with their corresponding 10 appliance operating statuses, as well as the scenario without any appliances, as shown in Table 1. We collect data in an open area (Figure 11a) and evaluate our system concerning various aspects: appliances with different orientations (Figure 12b), appliances at different AoAs (Figure 12c), appliances at different distances (Figure 12d), varying quantities of appliances, and the presence of human interference. Furthermore, we gather data in a resting area (Figure 11b) to simulate real-world testing conditions. Our training data are collected for each appliance in a fixed position (Figure 12a), with 30 training samples per appliance operating status. Similarly, we collect 30 test samples for each specific condition during testing.

It should be noted that our testing data and training data are collected in different environments and on separate days. In both human-interference tests and real-world scenarios, there is a significant portion where the positions of moving individuals coincide with being at the same distance from electrical appliances.

**Metric:** We adopt accuracy as the detection metric, defined as the ratio of correctly classified samples to the total number of samples. In scenarios involving the detection of multiple objects, assuming that there are *n* data segments, that the *i*-th segment contains $m_{1}^{i}$ objects, and that, out of these, $m_{2}^{i}$ objects are accurately detected, the overall accuracy is calculated as follows:

(22) $$Acc=\frac{\sum_{i=1}^{n}m_{2}^{i}}{\sum_{i=1}^{n}m_{1}^{i}}.$$

### 4.2. Overall Performance

**Figure 13:**
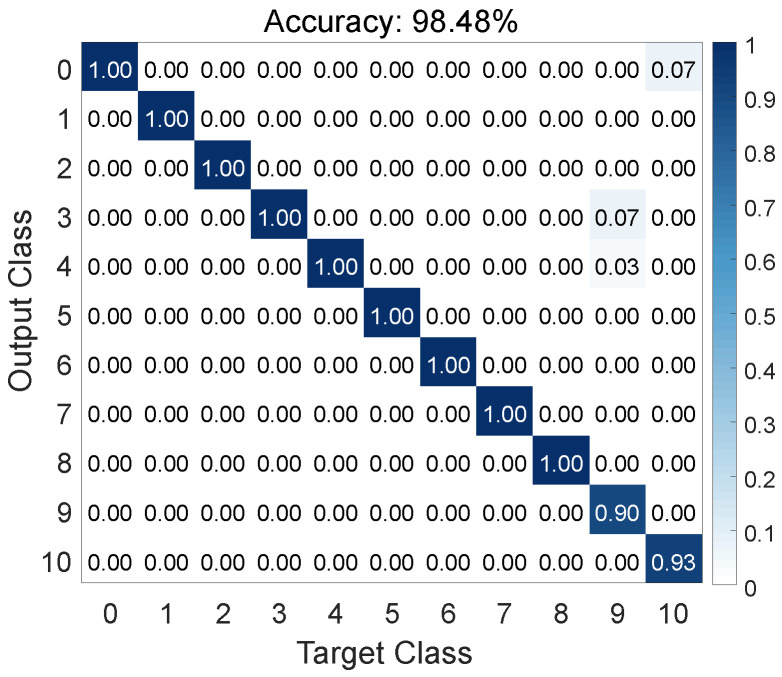
Overall performance of HomeOSD.

In this experiment, the tested electrical appliances remain in the same positions as during the training phase. We conduct tests for the 11 conditions listed in Table 1. Figure 13 illustrates the overall performance results. The overall recognition accuracy reaches 98.48%, demonstrating the effective capability of HomeOSD in detecting the operating status of appliances.

Within the confusion matrix depicted in Figure 13, it is evident that the majority of appliance operating statuses achieve a recognition accuracy of 100%. However, the recognition accuracy for the washing machine’s washing status is notably lower, at 90.00%. This is because, during the washing process, the motor of the washing machine operates in a variable-speed state.

### 4.3. Effectiveness of the Proposed Design

In this section, we conduct tests to evaluate how the newly designed Vibration Intensity Metric and the AWMDC classifier contribute to system performance improvement.

**Benefits of Vibration Intensity Metric for Appliance Distance Estimation.** In previous works, two common methods were used to estimate the distance of vibrating objects with mmWave radar: the Doppler-based method and the symmetry-based method. The Doppler-based method estimates distance based on intensity in the Doppler-FFT, while the symmetry-based method leverages the back-and-forth motion of vibrations and considers Doppler effects symmetrically in both the forward and reverse directions. However, these methods often fail in real-world scenarios due to Doppler effects from human activities. Therefore, we use the difference in distance estimation for the same vibrating appliance with and without human presence as an error metric to evaluate and judge the effectiveness of different methods for appliance distance estimation.

**Figure 14:**
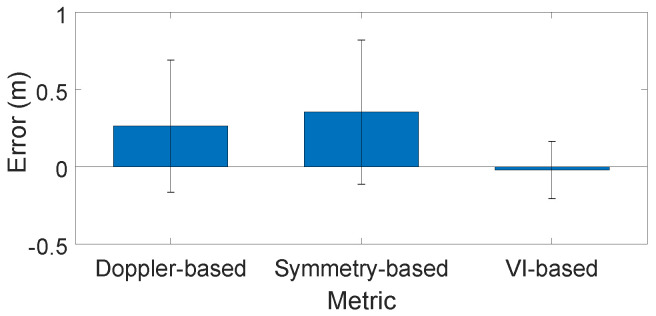
Error for different distance estimation methods.

Figure 14 presents the results for different methods, with mean errors of 0.26 m for the Doppler-based method, 0.35 m for the symmetry-based method, and −0.02 m for the Vibration Intensity-based method. The standard deviations of errors are 0.43 m, 0.47 m, and 0.19 m, respectively. The results indicate that the Vibration Intensity-based method outperforms the others in terms of both precision and stability, highlighting its resilience to the influence of human activity.

**Figure 15:**
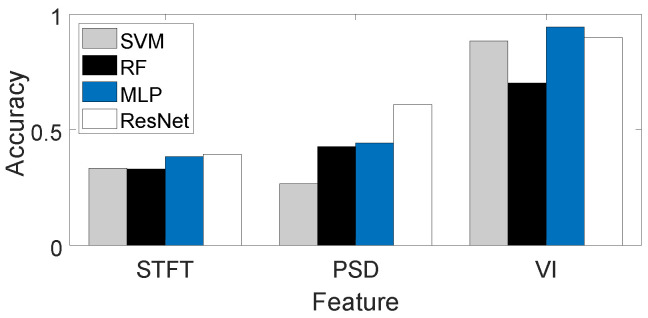
Recognition accuracy using different features.

**Benefits of Vibration Intensity-Based Feature.** Short-Time Fourier Transform (STFT) and Power Spectral Density (PSD) are common frequency features, and we compare these two features with Vibration Intensity (VI). We feed these three types of features separately into common classifiers and calculate classification accuracy. The classifiers used include Support Vector Machine (SVM), Random Forest (RF), Multi-Layer Perceptron (MLP), and Residual Neural Network (ResNet). The classification results for different features are shown in Figure 15, where the recognition accuracies using STFT and PSD as features are below 70%, while the recognition accuracies using Vibration Intensity as a feature exceed 70%. This demonstrates that Vibration Intensity is more effective than other features.

**Figure 16:**
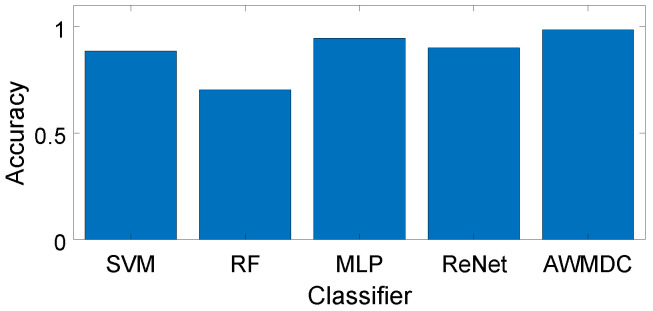
Recognition accuracy using different classifiers.

**Benefits of the AWMDC:** We compare the recognition accuracy and training time of our designed AWMDC with other classifiers. As shown in Figure 16, the recognition accuracies for SVM, RF, MLP, ResNet, and AWMDC are 88.48%, 70.30%, 94.55%, 90.00%, and 98.48%, respectively. Training times are provided in Table 2. It is evident that the AWMDC classifier not only achieves the highest recognition accuracy, but also boasts a very short training time.

In practice, it is essential for classifiers to have short training times, as households often introduce new appliances. While SVM and RF have shorter training times, their classification accuracy is insufficient. MLP and ResNet offer classification accuracy similar to AWMDC but come with significantly longer training times. Overall, AWMDC emerges as the optimal classifier.

**Benefits of Introducing Coefficients.** To assess the influence of introducing coefficients on detection performance, we provide a table demonstrating the system’s recognition accuracy under different values of relaxation and constant weighting coefficients, as depicted in Table 3.

It is crucial to clarify that, when the absolute maximum value of the relaxation coefficient is 0, this essentially indicates the absence of a relaxation coefficient. Similarly, setting the constant weighting coefficient to 1 signifies the non-utilization of such a coefficient. Table 3 demonstrates that, without employing either the relaxation or constant weighting coefficients, the recognition accuracy is 96.67%. However, upon integrating both coefficients, the maximum achievable recognition accuracy increases to 98.48%. This table effectively validates the effectiveness of these two coefficients in our system.

### 4.4. Different Micro-Benchmarks

We undertake a detailed exploration of a series of micro-benchmarks to provide a comprehensive understanding of HomeOSD’s performance.

**Figure 17:**
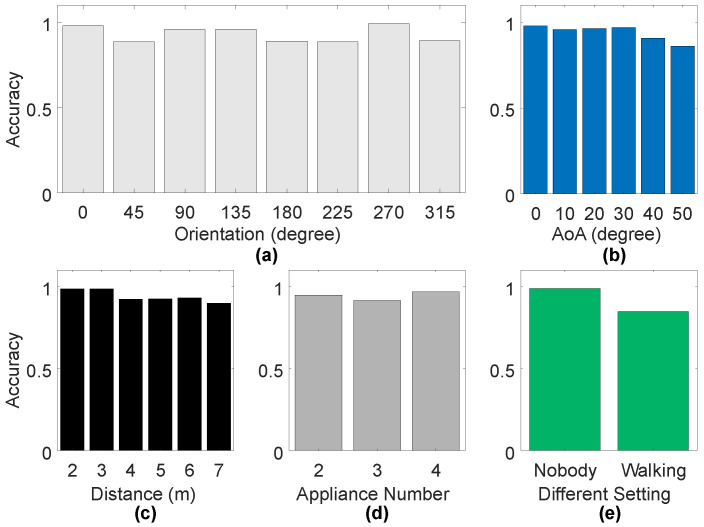
Performance of different micro-benchmarks. (**a**) Performance with different orientations; (**b**) Performance under different AoAs; (**c**) Performance under different distances; (**d**) Performance under different numbers of appliances; (**e**) Performance under the interference of walking.

**Performance with Different Orientations:** We maintain the positions of the appliances while altering their front-facing orientations, as illustrated in Figure 12b. The tested orientations include 0 degrees, 45 degrees, 90 degrees, 135 degrees, 180 degrees, 225 degrees, 270 degrees, and 315 degrees. The corresponding recognition accuracies are 97.88%, 88.48%, 96.76%, 95.76%, 88.79%, 88.48%, 99.09%, and 89.09%, as shown in Figure 17a. These results indicate that, even with changes in the reflective surfaces of the appliances, our system maintains a consistently high level of recognition accuracy.

**Performance under Different AoAs:** We position the appliances at different angles of arrival (AoAs), as depicted in Figure 12c. The recognition accuracies for appliances at AoAs of 0 degrees, 10 degrees, 20 degrees, 30 degrees, 40 degrees, and 50 degrees, as shown in Figure 17b, are 97.88%, 95.76%, 96.36%, 96.97%, 90.61%, and 86.06%, respectively. We observe a slight decrease in recognition accuracy as AoA increases, but it remains at a high level within the radar’s Field of View (FOV).

**Performance under Different Distances:** We increase the distance of the appliances from the radar from 2 m to 7 m, as depicted in Figure 12d. The recognition accuracies at different distances, shown in Figure 17c, are 98.48%, 98.48%, 92.12%, 92.42%, 93.03%, and 89.70% for distances from 2 m to 7 m. Most room lengths do not exceed 7 m, so our system is generally effective in typical scenarios.

**Performance under Different Numbers of Apliances:** We test the system’s performance when multiple appliances are operating simultaneously. To mitigate interference from appliances with larger vibration amplitudes on those with smaller amplitudes, we implement a sliding window operation with a 10 s duration, as detailed in Section 3.4.2 of the field. The results, shown in Figure 17d, indicate recognition accuracies of 94.69%, 91.43%, and 96.73% for two, three, and four appliances, respectively. These experiments demonstrate that HomeOSD can effectively detect the operating status of multiple appliances simultaneously.

**Performance under the Interference of Walking:** To validate HomeOSD’s ability to suppress interference caused by human activity, we test the system’s recognition accuracy when people are walking around in the vicinity. The result, as shown in Figure 17e, yields a recognition accuracy of 84.55% when there is human activity nearby. This indicates that our system can effectively mitigate interference from surrounding human activity.

### 4.5. Performance in the Real World

To further assess the practical application of our system, we conduct experiments in a real-world environment, as illustrated in the deployment setup shown in Figure 11b. We test the recognition accuracy under three scenarios: no individuals present, individuals seated, and individuals walking. It is worth noting that, to mitigate any potential blockage of mmWave propagation between appliances and the radar during human movement, we employ a 10 s sliding window during the recognition process, as described in Section 3.4.2 of the field.

**Figure 18:**
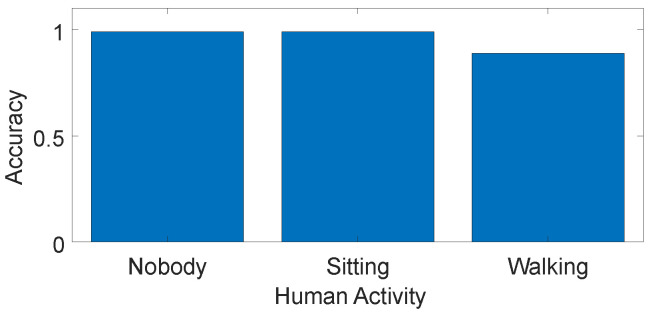
Performance in real-world scenarios.

The results, as depicted in Figure 18, indicate recognition accuracies of 98.98%, 98.98%, and 88.78% for the three scenarios, with an average accuracy of 95.58%. These findings highlight the robust performance of HomeOSD in real-world settings.

## 5. Discussion

**Add New Appliances:** As shown in Table 2, our classifier merely requires less than a minute of data collection for a new appliance and just a few seconds for training. Moreover, the number of appliance operating statues to be detected within a single room typically does not exceed 10. Thus, our system is well-equipped to handle the addition of new appliances.

**Transportability:** Currently, mmWave radar technology finds diverse applications in human-activity detection, including gesture recognition, trajectory tracking, gait detection, skeleton detection, and so on. HomeOSD effectively categorizes appliance operating status independently of human activities, utilizing only data processing while maintaining the mmWave radar hardware. Consequently, the HomeOSD can seamlessly integrate with existing indoor mmWave radar systems without modification.

**Localization of Appliances in 2-D:** HomeOSD possesses the capability to concurrently detect the operating statuses of appliances situated at varying distances. Nevertheless, under certain exceptional circumstances, multiple appliances may be equidistant from the radar, resulting in HomeOSD’s inability to differentiate them simultaneously. In research based on FMCW radar, established methods exist for target-angle detection and acquiring the 2-D position of targets. These algorithms can extract IQ domain signals at each 2-D position, rendering HomeOSD’s solution directly applicable for 2-D localization.

**Non-Line-of-Sight Detection:** HomeOSD relies on capturing mmWave signals that are directly reflected by appliances. When an appliance is obstructed by other objects, HomeOSD cannot detect its status through Line of Sight (LOS). To address this limitation, mmWave radar can be employed to detect appliance vibrations even in Non-Line-of-Sight (NLOS) scenarios. Numerous prior studies explore the use of walls as reflective surfaces for NLOS detection, including vibration sensing. Consequently, our forthcoming research will investigate methods to enhance NLOS detection capabilities within the HomeOSD system.

**Limitations:** While we demonstrate that HomeOSD can effectively detect the operating status of multiple appliances in real-world scenarios, it still has limitations in the following cases:

1. When appliances and the radar are situated in different rooms, mmWave signals may struggle to penetrate all walls, hindering the measurement of appliance vibrations. Therefore, a challenge remains in ensuring that a single radar can simultaneously detect all appliances throughout an entire house;
2. In the home, there are many devices without constant vibration frequency, such as a faucet. Utilizing HomeOSD to detect devices with irregular vibration periods poses a significant challenge.

To address these limitations, our future work will explore methods to detect the status of devices in other rooms by leveraging wall vibrations. For devices lacking specific vibration frequencies, such as faucets, we may employ transfer learning techniques to detect device status in diverse environmental conditions.

## 6. Conclusions

In this paper, we introduce HomeOSD, a contactless system built on mmWave radar technology. HomeOSD excels at simultaneously detecting the operating status of multiple appliances without interference from human activity. We propose an innovative metric called Vibration Intensity that leverages the periodic characteristics of vibrations, demonstrating resilience to interference from surrounding human activities. Additionally, we design an AWMDC to identify appliance operating status, and it remains robust against fluctuations in appliance vibrations. Remarkably, our system achieves an impressive detection accuracy of 95.58% in real-world scenarios, even with a limited amount of training data. We believe that HomeOSD can leverage mmWave radar technology to efficiently detect the operating status of multiple appliances in smart-home environments. Importantly, it can seamlessly integrate with existing mmWave radar systems without requiring hardware modifications.

## Figures and Tables

**Table 1:** All appliances’ operating statuses.

Appliance	None	Air Conditioner			Fan			Microwave	Fridge	Washing Machine	
Operating status	NA	L ^1^	M ^2^	H ^3^	L	M	H	On	On	Washing	Drying
Label	0	1	2	3	4	5	6	7	8	9	10

^1^ Low speed. ^2^ Medium speed. ^3^ High speed.

**Table 2:** Training time for all classifiers.

Classifier	SVM	RF	MLP	ResNet	AWMDC
Training time (s)	0.01	0.09	1386.32	3506.68	3.75

**Table 3:** Performance of different introducing coefficients. Coefficient-free result is underlined. Best results are **bold**.

Constant Weighting Coefficient	Relaxation Coefficient				
	|μ|≤0	|μ|≤1	|μ|≤5	|μ|≤10	|μ|≤20
$\omega_{0}=0.1$	96.67%	97.27%	97.58%	97.58%	97.88%
$\omega_{0}=1$	96.67%	97.27%	97.58%	97.58%	97.88%
$\omega_{0}=5$	97.27%	97.27%	98.18%	98.18%	**98.48%**
$\omega_{0}=10$	97.27%	97.58%	98.18%	**98.48%**	98.18%
$\omega_{0}=100$	95.76%	93.94%	94.55%	**98.48%**	94.24%
$\omega_{0}=1000$	92.42%	92.12%	93.33%	72.73%	72.12%

## Data Availability

The data presented in this study are available on request from the corresponding author.
